# Role of basophils in the pathogenesis of minimal change nephrotic syndrome: A literature review

**DOI:** 10.3892/etm.2014.1901

**Published:** 2014-08-12

**Authors:** QINGJUN PAN, JING WU, JINGLI TAO, YANWEN CHEN, LU LI, ZHENZHEN DENG, WEIJING LIU, HUAFENG LIU

**Affiliations:** Institute of Nephrology, Affiliated Hospital of Guangdong Medical College, Zhanjiang, Guangdong 524001, P.R. China

**Keywords:** minimal change nephrotic syndrome, T helper cell 1, T helper cell 2, basophils, immunoglobulin E, Toll-like receptor

## Abstract

A number of studies have verified that minimal change nephrotic syndrome (MCNS) may result from the dysfunction of T cells and B cells, although the precise mechanisms are yet to be elucidated. It is widely recognized that MCNS is a T helper (Th)2-dominant glomerular disease caused by an imbalanced Th1/Th2 immune response. Increased levels of the Th2 cytokines, interleukin (IL)-4 and IL-13, have been demonstrated to be closely associated with disease activity. In addition, basophils can affect the Th1/Th2 balance by enhancing the Th2 response and impairing the Th1 response, which are then involved in the development of numerous diseases. However, whether basophils are vital in the pathogenesis of MCNS remains unknown. Frequent positivity of the human basophil degranulation test in patients with MCNS has been observed. Thus, basophils should be analyzed in order to determine their role in the pathogenesis of MCNS.

## 1. Introduction

Minimal change nephrotic syndrome (MCNS) is an idiopathic nephrotic syndrome that is common among children. The condition is characterized by detectable, glomerular podocyte foot process fusion without evident kidney lesions, and is defined by proteinuria of >3.5 g/day and hypoproteinemia of <30 g/l. The levels of edema and hyperlipidemia vary between individuals. MCNS typically occurs in children and is particularly sensitive to corticosteroid treatment, where a short duration of remission and a frequent rate of relapse are often observed (1). However, the mechanisms of MCNS are not fully understood. Certain studies have proposed that MCNS is a T helper (Th)2-dominated disease that may be associated with a circulating factor from MCNS T cells (2,3).

CD4^+^ T cells are categorized on the basis of their distinct cellular functions and the cytokines secreted. At least two subsets are involved in the development of MCNS: Th1 cells that are characterized by the secretion of interferon-γ and tumor necrosis factor-β, and Th2 cells that are identified by their secretion of the proinflammatory cytokines, interleukin (IL)-4, IL-5 and IL-13, which are associated with the pathogenesis of immunoglobulin E (IgE) and eosinophilia. Previous studies have indicated that MCNS is associated with atopy (4–7), as well as increased levels of IgE (8–12), IL-13 and IL-4 (13–16).

Basophils are derived from marrow hemopoietic stem cells, and constitute ≤1% of the leukocytes in the peripheral blood (17). Basophils express a high-affinity IgE receptor (FcɛRl); thus, are associated with IgE-mediated allergic inflammation and innate immunity. Following stimulation, basophil granules release numerous substances, including histamine, heparin and slow-reacting substances of anaphylaxis, which results in the mediation of anticoagulation, the alteration of vascular permeability, the contraction of smooth muscles and hypersensitivity. Previously, the functions of basophils have been developed *in vivo* and *in vitro*; with this cell type recognized as the original source of IL-4 and the cause of Th2 polarization induction. However, the precise mechanism underlying the functions of basophils *in vivo* remains poorly understood.

Therefore, the present study investigated whether an association exists between MCNS and basophils. Possible new perspectives for understanding the role of basophils in the pathogenesis of MCNS were summarized in the present study.

## 2. Imbalance of the Th1/Th2 response and the role in MCNS

The etiology of MCNS is complicated and elusive. Infections and atopy are commonly considered to induce patients with MCNS to relapse in clinics. Increasing evidence has demonstrated that the aberration of T cells may play a crucial role in the pathogenesis of MCNS, and the importance of T cells in MCNS was first proposed by Shalhoub in 1974 (18). Accordingly, the hygiene hypothesis (19) proposed that MCNS was a Th2-predominant immune response. The balance of the Th1/Th2 response, particularly during childhood development, may be responsible for the later development of specific human glomerulonephritis (GN) (20). A number of studies have observed elevated mRNA expression levels of Th2 cytokines in the peripheral blood leukocytes of MCNS patients (21–24). In addition, a promising but unconfirmed glomerular permeability factor (GPF) from T cells may be essential to the development of MCNS (25). When this factor was injected intravenously into rats, significant proteinuria was induced and partial fusion of the glomerular epithelial cells was observed via electron microscopy (25). Growth regulated protein (GRO)-γ is a potential candidate for the Th2-associated GPF in MCNS, as implicated in the function of endothelial cells. Furthermore, Adrogue *et al* (26) observed markedly higher levels of CD4^+^ T cells in patients with MCNS and hypothesized that the levels of IL-4, IL-8 and GRO were also higher in individuals with MCNS. An increase in the level of IL-8 was shown to change the permeability of the glomerular basement membrane (GBM) via reducing the synthesis of heparan sulfate proteoglycans (HSPGs) on the GBM, which eventually induced proteinuria in rats (27). This observation indicated that patients with MCNS tended to develop a Th2-dominant T cell response.

Receptors for IL-4 and IL-13 are also present in podocytes (28,29), and previous studies have detected higher levels of serum IL-13 and IL-4 in patients with MCNS (13–16). In an animal model, the overexpression of IL-13 induced minimal-change-like nephropathy in rats (30). Furthermore, an additional study demonstrated that IL-13 induced podocyte injury via a signal transducer and activator of transcription-6-dependent pathway in the podocytes of mice (31). Triptolide was able to protect podocytes from IL-13-induced injury *in vitro* (32). However, the effect of IL-4 on podocyte injury is not well understood and requires further study in the future.

Th2 responses are characterized by IL-4, IL-13 and other Th2 cytokines, which trigger the switch from immunoglobulin M (IgM) to IgE production in B cells. Elevated levels of IL-4 and IL-13 contribute to the progression of Th2-type disease by blocking the differentiation of naive T cells into Th1 cells. Thus, immune dysregulation plays a crucial role in the pathogenesis of MCNS, although the precise mechanisms remain unclear. Further study concerning the dynamics of the Th1/Th2 response in MCNS may aid the understanding of the disease and be useful for the prevention, treatment and prognosis of MCNS.

## 3. Role of basophils in the dynamics of Th1/Th2

Basophils are primary effector cells involved in IgE-mediated allergic inflammation and innate immunity (33). These cells play distinct roles in allergic inflammatory disease (34); however, further verification of this has remained elusive until recently. Basophils are able to induce the development of Th2 cells *in vitro* and *in vivo* (35), and the depletion of basophils using antibodies against FcɛRl has been shown to diminish the development of Th2 cells (36). Furthermore, following the cross-linking of FcɛRl-bound IgE by multivalent antigens, basophils can rapidly produce diverse mediators, such as the cytokines IL-4 and IL-13. As basophils are the prime early producers of IL-4, Th2 cytokines, which promote naive CD4^+^ T cell polarization, trigger the differentiation of Th2 cells and support humoral memory responses (37,38). Thus, basophils play an essential role in Th2 differentiation from naive CD4^+^ T cells, which are also dominant in the pathogenesis of MCNS.

## 4. Basophils may be involved in the pathogenesis of MCNS by affecting the dynamic balance of Th1/Th2

Hypotheses involving basophils in idiopathic nephrotic syndrome (INS) can be traced back to Pirotzky *et al* in 1982 (39). In the study, the human basophil degranulation test (HBDT), which assumes that the degranulation of basophils in the presence of a specific allergen is an index of an IgE-dependent cellular response, was tested on 46 unselected patients with INS (28 with MCNS and 18 with focal segmental glomerular sclerosis) with or without atopic manifestations. Of the patients with MCNS, 57% (16/28) had a positive result and were accompanied by basophil activation (39). Laurent *et al* also observed frequent positivity of the HBDT in individuals with INS (minimal glomerular changes and segmental and focal glomerulosclerosis) (40). Furthermore, Mack and Rosenkranz stated that basophils, which are not frequently studied, should be analyzed when investigating the pathogenesis of MCNS (3).

**Figure 1:**
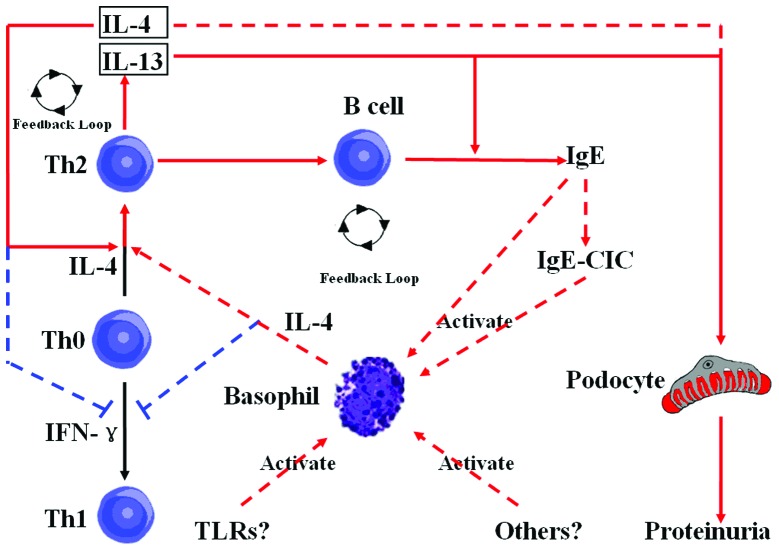
Proposed interactions between basophils, B and T cells in individuals with minimal change nephrotic syndrome (MCNS). The red and black solid lines with arrows represent interactions that have been previously reported, while the red and blue dotted lines with arrows represent interactions that have not been reported in MCNS. The red lines with arrows represent promotional functions and the blue dotted lines with flat heads represent suppressive functions. IL, interleukin; Th, T helper cell; TLR, Toll-like receptor; IFN, interferon; IgE, immunoglobulin E; CIC, circulating immune complexes.

Based on the evidence that MCNS is a Th2-dominant immune response, basophils, as promoters of the Th2 immune response and crucial sources of IL-4, which may initiate the disease, play an important role in maintaining a Th2-immune response and abating the Th1-immune response, resulting in the aberration of T-cell function. Thus, basophils may be involved in the mechanism of MCNS via affecting the dynamic balance of the Th1/Th2 response (Fig. 1).

## 5. Mechanisms of basophil activation in patients with MCNS

A number of major pathways may be involved in the activation of basophils in MCNS.

### IgE-circulating immune complexes (IgE-CIC) mediate basophil activation

Previous studies have demonstrated that serum levels of IgE are significantly increased in patients with MCNS, and high levels of serum IgE may be associated with poor prognosis (10,41,42). However, IgE or IgE-CIC have not been identified in renal tissue and no experimental models of IgE-mediated nephropathy have, to the best of our knowledge, been reported. Thus, this evidence indicates that the elevated levels of serum IgE observed in MCNS may reflect the abnormal regulation of IgE synthesis or B or T cell activation, which reflects immune dysfunction, but not a role in the pathogenesis of MCNS.

Through the use of a combination of CIC assays, Cairns *et al* observed CICs in the majority of the 271 serum samples collected from 131 patients with idiopathic MCNS, membranous and mesangial proliferative GN (43). Thus, high serum levels of IgE may mediate basophil activation in individuals with MCNS. Furthermore, autoreactive IgE immune complexes were shown to activate peripheral basophils in a mouse model of lupus nephritis (44). Basophils express the high-affinity IgE receptor, FcɛRl (45), which cross-links with IgE to activate a series of signaling molecules, including Fyn, Syk, Lyn, phosphoinositide 3-kinase and Akt, which subsequently induces basophil activation and results in the release of inflammatory mediators that are able to mediate tissue injury (46–48). In addition, the levels of a series of surface molecules, including CD203c, CD63 and CD11b, can significantly increase and rapidly produce a large quantity of cytokines, including IL-4 and IL-13. IgE synthesis by B cells requires two signals, one delivered by IL-4 or IL-13, which are released by Th2 cells, and the other via the interaction between the CD40 receptor on B cells and the CD40 ligand expressed by activated T cells. Activated basophils are able to produce IL-4 and IL-13 and also express the CD40 ligand, through which they are able to interact with B cells. Furthermore, the production of IL-3 by basophils may switch the Ig type towards IgE, which in turn may activate basophils. In this context, autoreactive IgE and activated basophils may be involved in the pathogenesis of MCNS by affecting the balance of the Th1/Th2 immune response.

### Toll-like receptors (TLRs) mediate basophil activation

A number of TLRs are expressed in basophils, including TLR2, TLR4, TLR9 and TLR10 (49). Peptidoglycan is a TLR2 ligand that activates basophils, resulting in the secretion of IL-4 and IL-13, which are independent of nuclear factor-κB activation (50). Basophils can secrete both IL-4 and IL-13 in direct response to peptidoglycan, and peptidoglycan can also augment the secretion of IL-4 and IL-13 in response to IgE-dependent activation, and the secretion of IL-13 in response to IgE-independent stimulation. Furthermore, the inhibition of nuclear factor-κB does not prevent these enhancing effects mediated by peptidoglycan. TLR4 expressed on basophils may be involved in the pathogenesis of the infection-induced exacerbation of allergic inflammation (49). However, the expression and function of TLR10 in humans and mice is inconsistent and controversial (51).

TLR9 has been found to be expressed in the intracellular compartments of basophils (52). A previous study revealed that TLR9 is involved in the activation of peripheral blood basophils in patients with systemic lupus erythematosus (53). Furthermore, in basophils obtained from patients with MCNS, higher expression levels of TLR9 were identified (data not shown), and KU-812 cells, a basophil cell line, was shown to be activated by TLR9 agonists (53). However, whether TLR9 mediates basophil activation of MCNS *in vivo* remains unclear. The negative separation of primary human basophils is required for further investigation.

## 6. Conclusion

In conclusion, following the activation of basophils by IgE complexes or TLRs, various mediators, including IL-4, IL-8, IL-13 and regulated on activation, normal T cell expressed and secreted protein, are released (54). These mediators promote the differentiation of naive Th cells by Th2 and may be involved in the pathogenesis of MCNS (55). Based on this evidence, the study of basophils in patients with MCNS may provide novel perspectives on the pathogenesis of the disease.
